# On the Effects of Arsenic in Three Cases of Eruption

**Published:** 1805-08-01

**Authors:** J. C. Otto


*On the Effects of Arsenic in three Cases of Eruption; communicated by* J. C. Otto, *to the Editor of the Philadelphia Medical Museum.*

Sir,

I WAS requested by a young gentleman, to give him some advice and medicine, on account of an eruption that appeared upon various parts of his system, but particularly his abdomen. He informed me, he had been subject to it about six months, and, upon the supposition that it was a trifling and accidental thing, and would disappear shortly, he had done nothing for it, except washing it with a solution of the acetite of lead.

It took place sometimes merely upon rubbing the skin, and would then rise in small lumps resembling mosquito bites, which in the course of the day would subside, except leaving a small vesicle filled with a thin whitish humour. By puncturing the cuticle this was discharged, and the part soon became perfectly well. Sometimes the eruptions would be circular, in the form of small ring-worms, with a space in the centre perfectly free from disease; at others, one small pimple would rise, and be attended with such an intolerable itching that it was impossible to refrain from scratching; in a day or two a number of similar ones would appear around it, occupying a surface as large as a cent. Every form of the eruption terminated in a day or two in vesicles containing a thin fluid, which in two or three more would burst, and the parts would then in a short time become entirely well. No one kind predominated or occupied particular parts, but they were interspersed among each other. At first the abdomen was the seat of the disease, but it gradually extended itself over the lower, and then the upper extremities, leaving the neck and face untouched. Occasionally it would nearly cease to exist upon an arm, a thigh, or a leg, for a week, and afterwards break out with its usual violence. No method of treatment, except the last, had any material influence upon it; the disposition to restoration, that would sometimes take place, appeared to be the effect of accident, for it was never permanent, and would discover itself in a degree, under every mode of cure, and even in the short intervals when no medicine was administered.

I directed the patient to keep his skin perfectly clean, and he has frequently informed me, that, while he was under der my care, he changed his linen very often, and washed himself at least twice a week, with Castile soap and water. He was requested to live rather abstemious in his diet, avoiding salted and highly spiced food, and spirituous liquors, and to take a purge twice a week. After persisting in this treatment during five weeks, he called upon me, and mentioned that the disease was no better, and that it began to spread in a considerable degree on various parts of him. I gave him some ungt. hydrarg. nitrat. and ordered it to be applied to the parts affected every evening. After using it for a length of time, without any benefit, it was discontinued, and a strong solution of corrosive muriate of mercury substituted. The result of this application was similar to the preceding, for although the eruptions occasionally disappeared, they still broke out on other parts, so as to exist in an increasing quantity constantly. He afterwards used a great variety of topical remedies with no better success, and at length became afraid that he was infected with the itch; but his alarms were partly quieted, by recollecting that he had not communicated it to any of the persons with whom he had slept. He was notwithstanding anxious for the exhibition of sulphur, and wished to be saturated with it, with which I complied, and directed him to use the ungt. sulph. in the usual manner, after he had taken half a drachm of the flowers daily for the space of a week, but he chose to continue the dose forty days. During a greater part of this period his perspiration was strongly impregnated with the medicine, so as to be disagreeable to himself and others; and finding his situation not to be improving, he relinquished taking it. For a small time he took nothing of any kind, but perceiving the disease to become more general over him, he became exceedingly anxious to have something effectual done, and was willing to submit to any thing that brought with it the hope of benefitting him. I now determined on a slight salivation, to which he readily assented, and preferred impregnating his system slowly, without producing much affection of his salivary glands and fauces. He took a small quantity of hydrarg. muriat. mit. every evening, and in about a fortnight from his commencing it, his mouth became sore, and a salivation ensued; this medicine was still continued in the same dose ten days longer, and then omitted. This treatment was not more effectual in removing his complaint than the former ones had been. The obstinacy of the disease increased his solicitude, and he determined to persevere in the use of medicines of some kind, until a cure could could be effected. I now directed him to take sulphurated antimony in pills, with a diaphoretic drink; which course he pursued six weeks, when he discovered its inefficacy and discontinued it. A vegetable diet was likewise of no benefit. The Lisbon diet-drink was next resorted to, and taken for three months under every favourable circumstance; but this remedy was not attended with more success than those previously employed. I had read of the influence of arsenic in diseases of the skin, and had witnessed it in two instances. Once in a species of herpes, and afterwards in a large old sore, whose venereal virus had been destroyed by mercury, but whose cure could not be effected by it. As many of the usual remedies had failed in this case, and the round of them nearly completed, I thought proper to resort to this powerful but safe medicine; and preferring Fowler's mineral solution, I ordered ten drops to be taken three times a day for a week. The eruptions then began to disappear fast, and no fresh ones succeeded; and after the disuse of the remedy for a few days, a dose or two were taken daily for another week and then discontinued entirely. The disease now entirely ceased, and the skin became perfectly pure and clear; the small yellow blotches which remained, where the eruptions had been, disappeared in a short time, upon the peeling off of the cuticle. It has been some years since this cure was effected, and my patient has continued ever since perfectly free from his former disagreeable complaint.

During my attendance upon the above person, I was applied to in a case of a similar nature, and prescribed some of the medicines employed in the preceding one, none of which in this instance had any effect, except the mineral solution and mercury. After the solution had been taken a short time, the eruptions entirely disappeared, but returned upon its being omitted. Being obliged to resort to mercury to remove another disorder which he had contracted, it was observed, that the eruptions were removed, after a salivation had taken place a short period, but broke out again when it ceased. Perceiving I was able to suspend the disease, by the use of the solution, I supposed, that, when administered a long time, it would even remove the disposition to it, epecially as some change might have been effected in his habit by the operation of the mercury. Accordingly I requested him to persevere in the use of it a considerable period. Finding himself again relieved for a longer space than he had ever been before, he imagined

H 2 he he was entirely well, and, contrary to my directions, laid aside the medicine, upon which the eruption again recurred. The yellow fever now occurred and induced him to leave the city, since which time I have never heard of him.

Some young ladies of the Friends Society, who devote much time in relieving the distresses of the poor, desired my attention to a woman, who suffered much from an affection of her face. She said it commenced in June 1799, and was attended with considerable ulcerations; that it varied during the remainder of the year, spreading occasionally over a part of the cheeks and nose, and then nearly disappeared in the ensuing winter. During this period she employed a great variety of remedies, which her friends and acquaintances warmly recommended, but to no purpose. In the spring the complaint increased; and with a view of being perfectly cured, she applied to a physician, who gave her eight small powders of a white appearance, of which she was to take one every week, and confine herself rigidly to a mild diet. The medicine vomited and purged her so severely, that after having taken five doses, she determined to omit them, being so much exhausted by their operation, that she was scarcely able to walk across the floor, and preferred, she said, to die rather than make use of the residue. Her fauces swelled, gums became spongy, and teeth loose, but no salivation ensued; when she discontinued them, her nose and cheeks were rather better, and became entirely well in the fall, remaining however a good deal swelled and of a fiery redness, similar to the appearance that sometimes occurs in the faces of those who have been intemperate in the use of spirituous liquors. Such was the aspect of her face that it was with reluctance she ever left her house, being so frequently exposed to the unpleasant remarks of those whom she would meet in the streets. She remained well one whole year, at the expiration of which she fell down stairs, and hurt her ancle to so great a degree as to occasion it to become ulcerated: when it was almost healed, her face broke out again, her palate became highly diseased, and several pieces of bone came away from the roof of her mouth. Her palate was improved, when I was requested to visit her, March 1802, but her situation in other respects was much more alarming, being infinitely worse than she had ever been; there were considerable ulcerations of her nose, both externally and internally, and a similar affection of her cheeks, occupying one half of the right one, and about one fourth of the the other, nor did her upper lip entirely escape. The sores were tolerably deep and white at the bottom, with edges that were turned up and hard; the inflammation around them extended to some distance, and was of a bright scarlet colour, attended with such excruciating pain as to deprive her almost entirely of rest. Her state was so deplorable that we were both apprehensive she would lose the greater part of her nose, nor could we form any opinion to what extent the ravages in other parts would proceed. A few of the sores had healed without any application whatever, but were succeeded by others, so that the complaint had considerably increased of late. She was ordered to dress the parts affected morning and evening with lint moistened with sweet oil, and to take eight drops of Fowler's mineral solution three times a day, in a little water. This treatment produced an immediate and rapid improvement in her situation, and was persisted in six weeks, when she became entirely well. Two years have now elapsed since the cure was effected, without the least return of the disease; nor have any ill consequences followed the long continued use of the medicine

